# Depressive symptoms and stress among nurses in the COVID unit: A 7‐month cohort study

**DOI:** 10.1111/jjns.12477

**Published:** 2022-02-17

**Authors:** Kenjiro Tsubono, Chikako Ikeda

**Affiliations:** ^1^ Department of Psychosomatic Medicine Tokai Central Hospital Gifu Japan

**Keywords:** COVID‐19, COVID unit, depression, nurses, stress

## Abstract

**Aim:**

Previous studies have reported high prevalence of depression and anxiety symptoms among frontline nurses caring for COVID‐19 patients. Yet, only a limited number of prospective studies examining nurses' mental health problems have been performed. The present study aimed to examine depressive symptoms and COVID‐19‐related stresses among nurses working specifically in a hospital COVID unit over a 7‐month period.

**Methods:**

In this study, depressive symptoms and COVID‐19‐related stresses of a total of 28 nurses who worked in the COVID unit were assessed using the Quick Inventory of Depressive Symptom (QIDS) and the Tokyo Metropolitan Distress Scale for Pandemic (TMDP) over the 7‐month period from December 2020 to June 2021.

**Results:**

The mean QIDS scores in all participants fluctuated during the study period, showing a high correlation with the reported number of new COVID‐19 cases in the region. The mean TMDP scores showed a gradual decline over the period. Scores on the social stress factor of the TMDP demonstrated a statistically significant correlation with the QIDS scores.

**Conclusions:**

The results indicate that the number of new COVID‐19 patients in the region is associated with depressive symptoms among nurses in the hospital COVID unit. Scores on the social stress factor in the TMDP are also correlated with depressive symptoms among nurses.

## 1 INTRODUCTION

The emergence of COVID‐19 in China at the end of 2019 has led to a global pandemic, causing drastic disruption to social, economic, and healthcare systems globally (C. C. Lai et al., 2020). This public health emergency placed a significant physical and mental burden on frontline healthcare workers exposed to COVID‐19 (De Brier et al., 2020). A considerable proportion of all medical personnel reported symptoms of depression and other psychological distress. A previous study in China revealed that 50.4% of frontline healthcare workers reported experiencing depression (J. Lai et al., 2020). Nurses directly involved in caring for and managing affected patients have reported the greatest levels of anxiety and depression among all healthcare workers (Luo et al., 2020). The main source of anxiety reported by nurses has been fear of getting infected or accidentally infecting others (Mo et al., 2020). Shanafelt et al. (2020) identified additional sources of concern among nurses: lack of personal protective equipment, lack of access to childcare facilities during the pandemic, and lack of accurate information about the disease itself.

Additionally, frontline nurses face social stressors in the form of deterioration of human relationships, job insecurity, and income reduction (Matsumoto et al., 2021). Some nurses have been encouraged by their families to take leave from work, and others have been prevented from working in multiple facilities, leading to increased financial burden. Social stress has negatively influenced frontline nurses' motivation to work, which might lead to an increase in voluntary absenteeism among them (Shiwaku et al., 2021). To comprehensively evaluate pandemic‐related anxiety and social stress among medical personnel, Shiwaku et al. (2021) developed a new scale, the Tokyo Metropolitan Distress Scale for Pandemic (TMDP). The TMDP consists of two factors: concerns about infection, and social stress factors (Shiwaku et al., 2021). The TMDP has been found to be a valid detector of depressive symptoms, and its reliability and validity have been established (Shiwaku et al., 2021). To comprehensively assess psychological stress of frontline nurses involved with COVID‐19 patients, another study with a valid scale similar to the TMDP, including inquiries about social stressors, will be necessary.

Although several studies have examined COVID‐19‐related stress among healthcare workers, most of them have been cross‐sectional studies, which collected data only once over a short period. Very few longitudinal studies are available that evaluate frontline nurses' mental health challenges over a longer duration. Sampaio et al. (2021) conducted a cohort study that surveyed depression, anxiety symptoms, and quality of sleep among nurses over time. The results reported that nurses experience the psychological adaptation phenomenon: a mild reduction in depression, anxiety, and stress scores as time passes (Sampaio et al., 2021). However, this survey was conducted only three times across a relatively short period, between 31 March 31 and 4 May 2020. To evaluate the change in frontline nurses' stress over time more effectively, a longitudinal cohort study with a longer observation period will be required.

To address the above‐mentioned issues, the primary purpose of the present study was to investigate the changes in frontline nurses' psychological symptoms over time using a valid, comprehensive scale measuring COVID‐19‐related stress. For this purpose, we performed a seven‐month cohort study using the Quick Inventory of Depressive Symptomatology (QIDS) and the TMDP to examine the changes in depression and stress symptoms among nurses working specifically in a hospital COVID unit. We also investigated the relationship between demographic factors and psychological symptoms among them.

## 2 METHODS

### 2.1 Participants and data collection procedure

Tokai Central Hospital is a public hospital in the city of Kakamigahara, which is located in southern Gifu prefecture in Japan. It did not have an infectious special unit before the COVID‐19 pandemic. A part of the orthopedic unit was converted into a COVID unit, with 16 beds prepared for COVID‐19 patients. The hospital has mostly accepted COVID‐19 patients with moderate levels of severity due to the limited number of high‐care unit beds. The number of beds in the COVID unit changes in coordination with the infection stage of the prefecture. When the stage level decreases, reducing the hospital bed occupancy rate in the region, the beds and the nursing staff in the COVID unit are also temporally reduced. The stage level is assessed based on several factors, including the hospital bed occupancy rate in the region, and the reported number of new COVID‐19 patients per 100,000 people in the prefecture.

This prospective cohort study was carried out from December 2020 to June 2021 to evaluate depression symptoms and COVID‐19‐related stress among nursing staff in the hospital COVID unit. Only registered nurses who worked in the COVID unit during the designated seven‐month period were included in the study. A total of 28 nurses were assigned to the COVID unit during this period. Sixteen of them moved back and forth between the COVID unit and other care units, depending on the infection stage level. The remaining 12 nurses worked only in the COVID unit, as core nursing staff, throughout the seven‐month study period, regardless of the infection stage levels. All 28 nurses were included in this study. The characteristics of the participants are listed in Table 1. Of all the participants (*N* = 28), 21 (75.0%) were women, and 7 (25%) were men. Overall, 18 (64.3%) participants reported that they lived with someone, as opposed to 10 (35.7%) who lived on their own.

**TABLE 1:** Characteristics of all the participants (*N* = 28)

	*N*	%
Age (in years)		
20–29	7	25.0
30–39	11	39.3
40–49	10	35.7
Sex		
Female	21	75.0
Male	7	25.0
Experience as a nurse (in years)		
0–9	12	42.9
10–19	9	32.1
20 years or more	7	25.0
Living with someone		
No	10	35.7
Yes	18	64.3

At the end of each month, a paper‐and‐pencil psychological test battery including QIDS and TMDP was distributed to each participant, and the questionnaires were subsequently collected. For the 16 participants who moved between the COVID and other care units depending on the stage levels, the questionnaires were administered only for the months when they worked in the COVID unit.

### 2.2 Psychological test battery

#### 2.2.1 Depression

A Japanese version of the QIDS self‐report was used to evaluate the degree of depression (Fujisawa et al., 2010). The QIDS is a 16‐item multiple‐choice, self‐report inventory, with points ranging from 0 to 3. By adding the scores of each component, we made an evaluation based on total points ranging from 0 to 27 (Rush et al., 2003). Scores of 5 or lower were defined as indicating no depression, scores from 6 to 10 indicating mild depression, 11 to 15 indicating moderate depression, 16 to 20 reflecting severe depression, and total scores greater than 21 indicating very severe depression (Rush et al., 2003). The QIDS has been well‐established as a valid tool for determining patients’ depression levels during and after treatment.

#### 2.2.2 COVID‐19‐related stress

To assess the COVID‐19‐related psychological stress of participants, we adapted the TMDP (Shiwaku et al., 2021). The TMDP is a nine‐item multiple‐choice, self‐report inventory with a total score ranging from 0 to 36; higher scores indicate greater levels of stress. Shiwaku et al. (2021) conducted a factor analysis of the nine questions, revealing two factors: concerns about infection, and social stress factors (five and four items, respectively). The concerns about infection factors include questions related to the fear of the unknown infectious disease and perceived uncontrollability of the infection. The social stress factors include the negative impact of the COVID‐19 pandemic on human relationships and financial burdens on medical personnel. The TMDP has been established as a valid detector of depressive symptoms and anxiety, and its reliability and validity have been demonstrated (Shiwaku et al., 2021).

### 2.3 Statistical analysis

The scores of QIDS and TMDP are expressed as mean (*M*) and standard deviation (*SD*). To examine the difference in these scores between the different demographic categories, the average scores in each demographic category were calculated using the initial month's data for each participant. Categorical variables were expressed as counts and percentages. The normality of distribution was assessed using the Shapiro–Wilk test. Differences in continuous variables were compared using Welch's *t* test. An analysis of variance (ANOVA) was performed to compare more than two groups. The correlation between QIDS and TMDP scores was assessed using Pearson's correlation coefficient. All statistical analyses were performed using SPSS version 27 (IBM Corp., Armonk, NY, USA). Each test was conducted at a significance level of *p* < 0.05.

### 2.4 Ethics statement

The study was conducted according to the latest version of the Declaration of Helsinki and was approved by the Institutional Review Board of Tokai Central Hospital. All participants provided written consent after the nature of the procedures had been fully explained.

## 3 RESULTS

Table 2 shows the comparison of mean QIDS and TMDP scores between the participants living alone and those living with someone. There were no statistically significant differences between the two groups on either scale, although the TMDP scores in participants living alone were slightly higher than in those not doing so. Table 3 compares the mean QIDS and TMDP scores among three groups of participants with different durations of work experience as a nurse. Although there were no statistically significant differences among the groups, the scores on both scales in the most experienced group (20 years or more) were the highest. In contrast, the least experienced group (1–9 years) reported the lowest QIDS and TMDP scores.

**TABLE 2:** Comparison of the mean QIDS and TMDP scores of two groups (living alone vs. living with someone)

	Living alone (*n* = 10)	Living with someone (*n* = 18)	*df*	*t*	*p*	Cohen's *d*
	*M* (*SD*)	*M* (*SD*)				
QIDS	4.6 (3.3)	5.4 (4.5)	23.72	−0.56	0.580	−0.20
TMDP (Concerns about infection)	9.7 (3.1)	8.4 (2.8)	17.02	1.07	0.301	0.44
TMDP (Social stress)	2.9 (2.6)	2.6 (2.1)	15.94	0.30	0.765	0.13
Total TMDP score	12.6 (5.3)	11.1 (4.2)	15.16	0.79	0.441	0.34

Abbreviations: *df*, degrees of freedom; QIDS, Quick Inventory of Depressive Symptomatology; *SD*, standard deviation; TMDP, Tokyo Metropolitan Distress Scale for Pandemic.

**TABLE 3:** Comparison of the mean QIDS and TMDP scores of the three groups with different durations of work experience as a nurse

	1–9 years (*n* = 12)	10–19 years (*n* = 9)	20 years or more (*n* = 7)	*F* (2, 25)	*p*	η^2^
	*M* (*SD*)	*M* (*SD*)	*M (SD)*			
QIDS	4.9 (4.6)	5.1 (3.8)	5.6 (4.2)	0.05	0.949	0.00
TMDP (concerns about infection)	8.2 (2.5)	9.0 (2.7)	10.0 (3.7)	0.89	0.425	0.07
TMDP (social stress)	2.1 (1.9)	2.4 (2.0)	4.1 (2.7)	2.13	0.141	0.15
Total TMDP score	10.3 (4.1)	11.4 (4.2)	14.1 (5.4)	1.69	0.204	0.12

Abbreviations: QIDS, Quick Inventory of Depressive Symptomatology; *SD*, standard deviation; TMDP, Tokyo Metropolitan Distress Scale for Pandemic.

Regarding the correlation between the scores of the QIDS and each factor of the TMDP, the QIDS scores were positively correlated with the social stress scores of the TMDP (*r* = 0.407, *p* = 0.032). Although the QIDS scores were positively correlated with the concern‐about‐infection scores of the TMDP and the total TMDP scores, the correlation was weak and not statistically significant (*r* = 0.141, *p* = 0.473, and *r* = 0.289, *p* = 0.136, respectively).

**FIGURE 1:**
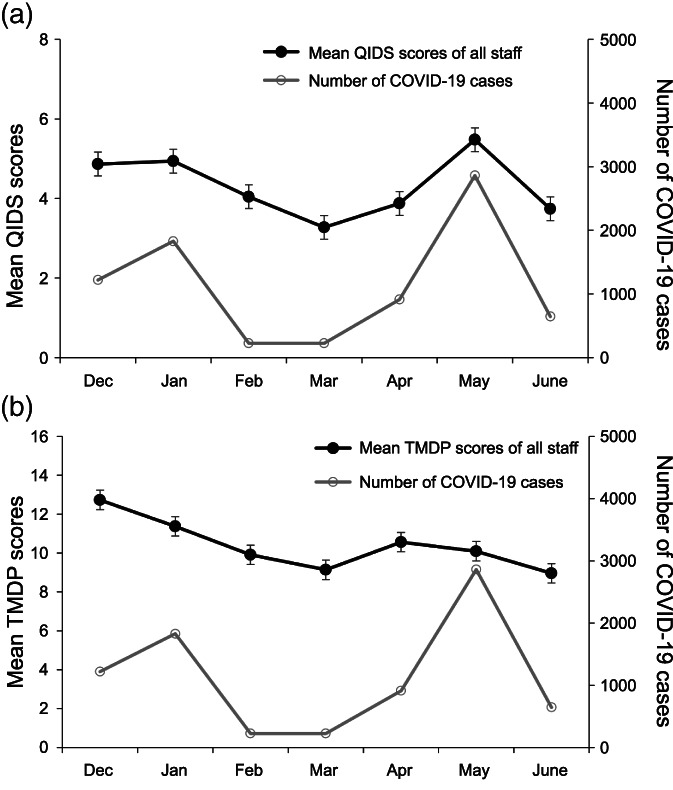
The change in mean (A) Quick Inventory of Depressive Symptomatology (QIDS), and (B) Tokyo Metropolitan Distress Scale for Pandemic (TMDP) scores of all nursing staff in the COVID Unit (*N* = 28) over the study period (from December 2020 to June 2021), with respect to the number of new COVID‐19 cases reported each month in the prefecture. Error bars represent standard errors

Figure 1 shows the change in mean QIDS and TMDP scores of all the participants (*N* = 28) over the seven‐month study period with respect to the number of new COVID‐19 cases reported each month in the prefecture. The QIDS ranged between 3.3 and 5.5 (keeping in mind the range of possible QIDS score is 0–27), indicating almost no depression (Fig. 1A). There were two peaks in the mean QIDS scores during January (score: 4.9) and May (score: 5.5). As the graph indicates, the mean QIDS scores of all participants and the reported numbers of new COVID‐19 cases in the prefecture highly correlated. Supporting the mean QIDS scores, numbers of new COVID‐19 cases in the prefecture were highest in January (1,828 cases) and May (2,860 cases). Pearson's correlation coefficient was used to analyze the correlation between mean QIDS scores and the number of new COVID‐19 cases in the prefecture, revealing a high correlation between them (*r* = 0.904, *p* = 0.005).

Figure 1B shows the change in the mean TMDP scores of all 28 participants over the study period. The mean TMDP scores ranged between 9.0 and 12.7 (keeping in mind the range of possible TMDP scores is 0–36). The highest score occurred in the initial month (December 2020). In April and May, when the reported number of COVID‐19 cases in the prefecture increased, the mean TMDP scores increased slightly as well. However, the scores generally decreased throughout the study period. There was no statistically significant correlation between the mean TMDP scores and the number of new COVID‐19 cases in the prefecture (*r* = 0.348, *p* = 0.445).

**FIGURE 2:**
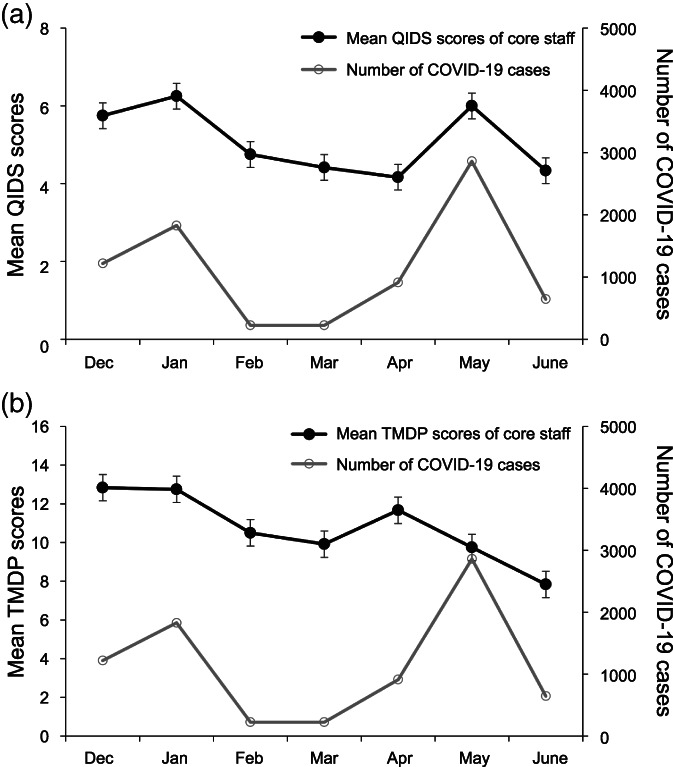
The change in mean (A) Quick Inventory of Depressive Symptomatology (QIDS), and (B) Tokyo Metropolitan Distress Scale for Pandemic (TMDP) scores of the core nursing staff in the COVID unit (*N* = 12) over the study period (from December 2020 to June 2021), with respect to the number of new COVID‐19 cases reported each month in the prefecture. Error bars represent standard errors

Figure 2 shows the change in mean QIDS and TMDP scores of the core nursing staff (*N* = 12), who worked only in the COVID unit throughout the seven‐month study period, with the number of new COVID‐19 cases in the prefecture. The QIDS scores ranged between 4.2 and 6.3, which were marginally higher than those of all 28 participants (Fig. 2A). There were two peaks of mean QIDS scores during January (score: 6.3) and May (score: 6.0), indicating mild depression in the core nursing staff. As shown in the graph, the mean QIDS scores of the core staff and the reported number of new COVID‐19 cases in the prefecture fluctuated simultaneously, revealing the same trend as did the analysis of all 28 participants' data. Pearson's correlation coefficient was used to analyze the correlation between the mean QIDS scores of the core nursing staff and the number of new COVID‐19 cases in the prefecture, demonstrating a statistically significant correlation between them (*r* = 0.787, *p* = 0.036).

Figure 2B shows the change in mean TMDP scores of the core nursing staff over the study period. The mean TMDP scores ranged between 7.8 and 12.8. The score in the initial month (December 2020) was the highest. In April and May, when the reported number of COVID‐19 cases in the prefecture increased, the mean TMDP scores marginally increased as well. Generally, the score decreased throughout the study period. There was no statistically significant correlation between the mean TMDP scores of the core staff and the number of new COVID‐19 cases in the prefecture (*r* = 0.200, *p* = 0.667).

## 4 DISCUSSION

The purpose of the present study was to examine the changes in depressive symptoms and COVID‐19‐related stress among nurses in the COVID unit over 7 months. The results showed that the mean QIDS scores of all 28 participants fluctuated during the study period. The QIDS scores increased in January (the second‐highest) and May (the highest), concurrent with increases in the reported number of new COVID‐19 cases in the prefecture. There was a high correlation between the mean QIDS scores and the number of COVID‐19 cases in the prefecture, revealing similar patterns of change over the study period. As the number of new COVID‐19 patients increased in the region, it was expected that the number of COVID‐19 patients requiring hospitalization would increase as well. It is considered that the stress levels of nursing staff in the COVID unit increased due to the increased number of patients and the high occupancy of the unit beds. This tendency was also shown in the mean scores of the core 12 nursing staff who worked only in the COVID unit throughout the study period. Although the mean QIDS scores of all the 28 participants indicated a no‐depression level (a score below 6.0), the scores of the core nursing staff indicated a level of mild depression (a score above 6.0). We suspect that working continuously in the COVID unit would be exhausting and stressful, causing psychological fatigue in nurses. However, the mean QIDS scores of the core nursing staff generally decreased over time. This trend contradicts the expected accumulation of psychological stress. This might be due to “the adaptation to the new normal,” as reported in a previous study (Zhang et al., 2020). A pattern of decline over time was seen in the mean TMDP scores, which reflect COVID‐19‐related stress, as well. The TMDP assesses concerns about COVID‐19 infection in medical personnel and its negative impact on their lives. It is possible that nurses in the COVID unit gradually adapted to the new normal situation over time, resulting in a decrease in concerns about COVID‐19 infection, and its related psychological strains in their lives.

Shiwaku et al. (2021), developers of the TMDP, indicated that this scale is correlated with a depression assessment scale, the Patient Health Questionnaire‐9 (PHQ‐9). In the present study, the total TMDP scores did not show a statistically significant correlation with the QIDS depression scores. The TMDP social stress factor scores, however, showed a statistically significant correlation with the QIDS scores. In contrast, the correlation between the QIDS scores and the concern‐about‐infection factors in the TMDP was not statistically significant. The TMDP social stress factors are those indicative of negative impacts on medical personnel's finances and their relationships with other people. This result suggests that the COVID‐19 pandemic has negatively affected a variety of social factors among frontline nurses, which might contribute to depressive symptoms and other psychological problems. Comprehensive social support for frontline nurses, which addresses relationship issues, social stigma, and financial insecurity, will be essential to reduce their mental stress.

Previous studies have reported that nurses with elderly family members exhibit high depression and anxiety scores compared with those who do not (Tercan et al., 2020). Matsumoto et al. (2021) reported that living with other people is a risk factor that increased social stress during the pandemic. In the present study, we analyzed the differences in depression and COVID‐19‐related stress symptoms between the two groups: those living alone and those living with someone. Although the difference was not statistically significant, contrary to the results of previous studies, the TMDP scores of participants living alone were slightly higher than those who did not. We did not specify ages or other characteristics of family members, as done in the previous study (Tercan et al., 2020), and the small sample size of the present study may have contributed to the inconsistent, statistically insignificant result. Additionally, we assessed the difference in the QIDS and TMDP scores among three groups categorized by years of nursing experience. The most experienced group (20 years or more) showed the highest score on both scales, indicating the highest psychological strain among the three groups. The nurses in the most experienced group were generally older than those in the other groups, making them statistically more vulnerable to COVID‐19 infection (Crimmins, 2020). This result was in accordance with that of a previous study, which reported that older hospital workers had a higher risk of depression, anxiety, and social stress (Matsumoto et al., 2021). Again, the sample size in the present study was small, which may not have been sufficient to generate a statistically significant result. It is a consistent tendency that advanced age increases the risk of psychological problems among frontline medical staff. The results suggest that the length of experience as a nurse does not contribute to the reduction of COVID‐19‐related stress.

The present study had some limitations. The main limitation was that the sample size was relatively small. The small sample size means that the study has low statistical power, which might have contributed to the lack of statistically significant differences in some variables. Additionally, we examined psychological stress among nurses in the COVID unit, where nurses primarily cared for patients with moderate severity levels. To evaluate stress among frontline nurses more comprehensively, it will be necessary to recruit participants in a variety of settings, for example, intensive care and pediatric units. Further, well‐designed, large, prospective trials will be required to address these issues.

Overall, given the association between the number of COVID‐19 new patients in the region and depressive symptoms among nursing staff in the hospital COVID unit, it is necessary to give them adequate organizational support particularly during the period of infection spread. The results also indicate that COVID‐19‐related social stresses are correlated with depressive symptoms among frontline nurses. Social stressors should be monitored and addressed continuously to reduce psychological stresses among nurses.

## 5 CONCLUSIONS

The study demonstrated that depressive symptoms among nurses in the COVID unit fluctuated over the seven‐month study period, but showed a gradual decline over time. Their QIDS scores showed a high correlation with the reported number of new COVID‐19 cases in the region, suggesting that psychological fatigue in the COVID unit staff was elevated as the number of COVID‐19 patients in need of hospitalization increased.

Although the total TMDP scores did not show a statistically significant correlation with the QIDS scores, among the two factors in the TMDP, the social stress factors scores showed a statistically significant correlation with the QIDS scores. This suggests that the deleterious effects of the COVID‐19 pandemic on human relationships and financial problems of COVID unit nursing staff might contribute to the symptoms of depression among them. Comprehensive social support is necessary to reduce the stress levels of frontline nurses.

## CONFLICT OF INTERESTS

All authors declare no conflicts of interest to disclose.

## AUTHOR CONTRIBUTIONS

Kenjiro Tsubono contributed to the design and implementation of the research, to the analysis of the results and to the writing of the manuscript. Chikako Ikeda critically reviewed the manuscript and supervised the whole study process. All authors critically revised the report, commented on drafts of the manuscript, and approved the final report.
